# Metabolic syndrome in patients with obsessive-compulsive disorder

**DOI:** 10.3389/fpsyt.2023.1164750

**Published:** 2023-08-15

**Authors:** Mohammadrasoul Khalkhali, Kiarash Rasekh, Fatemeh Eslamdoust-Siahestalkhi, Hassan Farrahi, Roghaye Zare

**Affiliations:** ^1^Kavosh Cognitive Behavior Sciences and Addiction Research Center, Department of Psychiatry, School of Medicine, Guilan University of Medical Sciences, Rasht, Iran; ^2^Neuroscience Research Center, Guilan University of Medical Sciences, Rasht, Iran

**Keywords:** obsessive-compulsive disorder, metabolic syndrome, obesity, body mass index, MetS

## Abstract

**Objective:**

Metabolic syndrome (MetS) is a collection of chemical and clinical risk factors. Patients with obsessive-compulsive disorder (OCD) might be at risk of MetS. This study aimed to investigate the prevalence and clinical correlates of MetS in an Iranian clinical sample of patients with OCD.

**Methods:**

We included 107 patients with OCD in a cross-sectional study. Demographic and clinical characteristics including OC symptoms, duration of treatment, age of onset, medications history, and comorbidity with other psychiatric disorders were collected.

**Results:**

The prevalence of MetS was 39.2%. Abdominal obesity was the most frequent component of MetS (68.2%), followed by low high-density lipoprotein cholesterol (50.5%). High serum triglycerides, high fasting serum glucose, high systolic blood pressure, and high diastolic blood pressure were observed in 47.7, 20.6, 18.7, and 9.3% of patients, respectively. Patients with MetS were older, married, had a low education level, had a high body mass index, and had no aggressive OC symptoms. MetS was not associated with psychiatric disorders comorbidities, age of onset, and duration of treatment.

**Conclusion:**

The results of this study were in line with the results of other studies that reported the poor health status of patients with OCD. A large number of patients are affected or are at risk of developing MetS. These patients need medical care along with the usual OCD treatments.

## Introduction

Obsessive-compulsive disorder (OCD) is a chronic and disabling disorder that affects ~2–3% of the population (1). The main symptoms are repetitive intrusive thoughts (obsessions) and distressing time-wasting rituals (compulsions) that are performed to get rid of obsessive thoughts (2). Selective serotonin reuptake inhibitors (SSRIs) and cognitive behavior therapy (CBT) are the most frequently used pharmacologic and psychotherapeutic treatments in patients with OCD (3, 4). OCD usually begins in adolescence period (5). Unfortunately, many of these patients have partial or poor responses to therapeutic interventions (6). It means that they have to take medications for most of their lives. Patients with OCD usually require higher doses of SSRIs compared to patients with depression or other anxiety disorders. The use of these agents and the time-wasting habits cause patients with OCD to pay less attention to their health and be at greater risk of weight gain, obesity, and metabolic syndrome (MetS) (6, 7).

MetS is a collection of chemical and clinical risk factors. It predisposes the patients to type 2 diabetes mellitus and cardiovascular and cerebrovascular events (8). The presence of MetS is associated with an increased incidence of chronic complications and mortality and has become a significant health hazard in the modern world. The global prevalence of MetS is estimated to be about one-quarter of the world population. It means that over a billion people worldwide are now affected by MetS (9). MetS includes abdominal obesity, abnormal glucose metabolism, elevated blood pressure, reduced high-density lipoprotein cholesterol (HDL-C), and atherogenic dyslipidemia (10). Three out of the five diagnostic criteria are needed for MetS diagnosis (10–14). Based on the criteria used for the definition of MetS, prevalence estimates vary. A national survey in Iran in 2007 reported that the prevalence of MetS was ~34.7% based on the ATP III criteria. This estimation was between 37 and 41% when using other criteria. The MetS was estimated to affect >11 million Iranians at the time of that study (15). Newly published studies indicated that MetS has remained a common and significant health problem in the Iranian population (16).

Along with the increasing concern about MetS in the general population, researchers have also paid more attention to MetS in patients with psychiatric disorders. The prevalence of MetS in patients with bipolar disorder, generalized anxiety disorder, depression, and schizophrenia has been reported to be higher than in the general population (17–21).

Despite numerous studies on other psychiatric disorders, it is surprising that only a tiny number of MetS studies have been carried out in patients with OCD. In a cohort study, Isomura et al. compared 25,415 patients with OCD with 12 million individuals from the Swedish general population and with their unaffected siblings. OCD was associated with an increased risk of metabolic or cardiovascular disorders in both comparisons. Patients with OCD were at risk for MetS that was not related to the dosage and duration of SSRI use (22). In another study carried out by the same group, there was an overall 25% increased risk of cardiovascular diseases. This risk was independent of the history of somatic illness, familial confounders, and other psychiatric comorbidities. The strongest association between OCD and cardiovascular disease was found for heart failure and venous thromboembolism (23).

In a cross-sectional Italian study by Albert et al., MetS was present in 21.2% of 104 patients with OCD, which was higher than its prevalence in the general Italian population. MetS was associated with cigarette smoking, absence or low physical activity, higher body mass index (BMI), and higher duration of exposure to antipsychotics. Long-term and high-dose medications, use of SSRIs and antipsychotics, early-onset of disorder, and high-risk lifestyle are factors that may put patients with OCD at risk for MetS (24). In a study by Aguglia et al. on 162 patients with OCD, 78 (48.1%) patients had at least one comorbid medical disorder. Endocrine/metabolic diseases (25.9%) were the most frequent general medical condition. The presence of a comorbid medical disorder was associated with the duration of untreated illness, older age, and lack of proper physical activity (25).

The relationship between OCD and general medical condition is less documented. To the best of our knowledge, only a few studies investigated the prevalence of physical health and MetS in patients with OCD. This study aimed to investigate the prevalence, sociodemographic, and clinical correlates of Mets in an Iranian sample of outpatients with OCD.

## Methods

One hundred seven outpatients with OCD admitted to two Guilan University of Medical Sciences supervised outpatient psychiatry clinics (Shafa university hospital and Dr. Khalkhali and colleagues) were recruited *via* convenience sampling from September 2020 to May 2021 and included in a cross-sectional study. The inclusion criteria were diagnosis of OCD, age ≥18 years old, score ≥ 16 (moderate and above severity) on the Yale-Brown Obsessive-Compulsive Scale (YBOCS) at the time of the first admission to the clinics, and at least 6 months of treatment and follow-up in the mentioned clinics. OCD and current comorbid psychiatric disorders were diagnosed using the Structured Clinical Interview for DSM-5 Research Version (SCID-5-RV) (26). The diagnosis of previous comorbid psychiatric disorder was made by referring to patients' files classified according to the DSM-4 or DSM-5 diagnostic criteria. Having one or more psychiatric disorders in the past or present time was defined as a comorbid disorder. Demographic characteristics and some clinical information were extracted from the patients' files. Additional necessary information was obtained by conducting clinical interviews, reviewing medical records, and interviews with family members. A board-certified psychiatrist carried out all the assessments, including the diagnosis of OCD and comorbid disorders, taking history, and rating YBOCS. The patients under corticosteroid treatment, pregnant women, and women who have just given birth were excluded. Current treatments for hypertension, diabetes or dyslipidemia, and corticosteroids were assessed by examining medical records and carrying out direct interviews with the patients. A senior medical student measured blood pressure, weight, height, and waist circumference in a well-equipped private room. He measured waist circumference at the level precisely between the lower border of the 12th rib and the upper border of the iliac crest. BMI was calculated as the ratio of weight (in kilograms) and height (in meters squared). We checked the patients' lipid profile and serum glucose every 6 to 12 months. Other physicians may also routinely monitor some of the patients. The results of previous blood tests in treatment follow-up ordered in our or other clinics were considered valid if they have not been taken more than 3 months ago. MetS was diagnosed according to the NCEP ATP III-modified criteria (NCEP ATP III 2002) as follows: systolic blood pressure ≥135 mm Hg or diastolic blood pressure ≥85 mm Hg or on antihypertensive medication, fasting serum glucose ≥100 mg/dL or on glucose-lowering medication, HDL ≤ 40 mg /dL in men and 50 mg / dL in women, and hypertriglyceridemia ≥150 mg/dl or on lipid-lowering medication. Waist circumference of ≥102 cm in men and 88 cm in women have diagnosed MetS if they had at least three out of five mentioned factors. The difference between YBOCS scores in the first and final assessment was indicated as the index of improvement.

### Sample size

The sample size was calculated according to the prevalence of MetS in patients with OCD in Isomura 's study (22), which was ~32%, based on Cochran's formula with a 95% confidence level and a 0.09 precision.

### Assessments

Yale-Brown Obsessive-Compulsive Scale (YBOCS). The YBOCS checklist was used to assess the OC symptoms types and severities and treatment follow-up. The severity of OCD is measured across five dimensions of obsessions and compulsions, namely time/frequency, interference, distress, resistance, and degree of control, based on a 5-point Likert (0–4) (27). Good reliability and validity were reported for this scale (28). In Iran, the internal consistency scores for the symptom checklist and severity scale were 0.97 and 0.95, respectively, and the test-retest reliability was 0.99 (29).

The Persian Version of the Structured Clinical Interview for DSM-5, Research Version (SCID-5, RV). The SCID-5, RV is a helpful tool to diagnose DSM-5 axis I disorders and for research purposes. The psychometric properties of SCID-5-RV showed an acceptable range for internal consistency (0.95–0.99), retest reliability (0.60–0.79), and Kappa reliability (0.57–0.72). In addition, the agreement between the interviewer and the psychiatric diagnosis was evaluated using the Kappa index, which resulted in a satisfactory result. In general, the Persian version of SCID-5-RV has good reliability and validity for different diagnoses in different clinical situations (26).

### Statistical analysis

Subjects' characteristics were summarized as mean ± standard deviation (Sd) for continuous variables and frequency (percentage) for categorical variables. The chi-square/Fisher's exact test was used to compare demographic and clinical characteristics between OCD patients with and without MetS. The Shapiro–Wilk test, plot test, and index of normality were used to determine the normality of the distribution. Continuous variables with normal distribution were summarized as median (interquartile range) and compared using an independent *t*-test. The comparison of continuous variables without normality was performed with the Mann–Whitney U-test. We used hierarchical logistic regression analyses with a MetS dummy variable as the dependent variable and significant demographic and clinical characteristics as predictors. This allowed us to address the question of what variables predict the MetS. According to the model fitting rule, non-significant variables were removed from a model by the backward LR procedure. The odds ratio of more than one shows more risk characterized as the MetS group. Alfa was set to 0.05. All data were analyzed using IBM SPSS Version 20.

### Ethics statement

The aims and procedures of the study were explained to participants who gave written consent. The ethics committee of Guilan University of Medical Sciences approved the study design (IR.GUMS.REC.1399.349).

## Results

The participants of this study were 107 OCD patients with a mean ± sd age of 40.93 ± 13.10 years. More than half of the patients (62.6%) were between 18 and 44 years old and the more frequent of them were women (74.8%). The mean ± sd of BMI was 28.62 ± 6.26 kg/m^2^. Table 1 shows the frequency of metabolic syndrome and its components. Abdominal obesity was the most frequent component of MetS (68.2%), followed by low HDL-C (50.5%). High serum triglycerides, high fasting serum glucose, and high blood pressure were observed in 47.7, 20.6, and 20.6% of patients, respectively. Only 19 patients (17.8%) had none of the MetS components. Forty-two patients (39.2%) met the criteria for MetS according to NCEP ATP III. The frequency of OCD patients who met four or five components was 26 (19.7%).

**Table 1:** Frequency of metabolic syndrome (MetS) and its components in OCD patients.

	**All patients (*n =* 107)**	**Cumulative frequency**
**Number of MetS component**, ***n*** **(%)**		
5^*^	5 (4.7)	5 (4.7)
4^*^	16 (14.9)	21 (19.7)
3^*^	21 (19.6)	**42 (39.3)**
2	24 (22.4)	66 (61.7)
1	22 (20.6)	88 (82.3)
0	19 (17.8)	107 (100.0)
**MetS component**, ***n*** **(%)**		
Abdominal obesity	73 (68.2)	-
Hypertriglyceridemia	51 (47.7)	-
Low HDL-C	54 (50.5)	-
High blood pressure	22 (20.6)	-
High fasting glucose	22 (20.6)	-

^*^According to the NCEP ATP III 2002 criteria, having at least three out of the five below components is a MetS diagnosed. Waist circumference ≥102 cm in men and 88 cm in women, hypertriglyceridemia ≥150 mg/dl or on lipid-lowering medication, high-density lipoprotein-cholesterol (HDL-C) ≤ 40 mg/dL in men and 50 mg/dL in women, systolic blood pressure ≥135 mmHg or diastolic blood pressure ≥85 mmHg on antihypertensive medication, fasting serum glucose ≥100 mg/dL or on glucose-lowering medication.

SSRIs were the most frequent medication (99.1%) followed by glutamatergic drugs (23.4%, three patients lamotrigine and 23 cases memantine) and second-generation antipsychotics (SGAs) (12.1%, aripiprazole and risperidone). Approximately 20.6% of patients were using other medications (17 patients buspirone and 5 patients clomipramine). Only three patients with bipolar disorder included in our sample were taking mood stabilizers. Approximately 55.1% of patients were treated with one drug, 34.6% with two drugs, and 10.2% with three drugs or more.

All demographic characteristics and their comparison between OCD patients with and without MetS are displayed in Table 2. Since very low frequency leads to insufficient statistical test power, comparisons were done based on the levels of categorical variables that had sufficient frequency. There was no significant difference between the distribution of gender and occupation between the two groups (*p* = 0.855, *p* = 0.144). The chi-square test showed that the prevalence of MetS in married patients was significantly higher than in single patients (*p* = 0.040). In patients with MetS, the mean education was significantly lower (*p* = 0.011) and the mean age and BMI were significantly higher (*p* < 0.0001, *p* < 0.0001) than in the patients without MetS (Table 2).

**Table 2:** Comparing demographic characteristics between OCD patients with and without metabolic syndrome.

	**All patients (*n =* 107)**	**Metabolic syndrome**		***x*^2^/ *t***	**df**	***p*-value**
		**Yes (*****n** =* **42)**	**No (*****n** =* **65)**			
Age (years)	40.93 ± 13.10	46.52 ± 12.74	37.3 ± 12.10	−3.768^+^	105	< 0.0001
**Age**, ***n*** **(%)**						
18–44	67 (62.6)	18 (47.4)	49 (76.6)	9.179	2	0.010
45–54	20 (18.7)	12 (31.6)	8 (12.5)			
55–64	15 (14.0)	8 (21.1)	7 (10.9)			
≥65	5 (4.7)	4 (9.5)	1 (1.5)	-	-	-
Education (years)	10.83 ± 5.21	9.33 ± 5.41	11.8 ± 4.89	973.5	-	0.011
**Sex, n (%)**						
Male	27 (25.2)	11 (26.2)	16 (24.6)	0.034	1	0.855
Female	80 (74.8)	31 (73.8)	49 (75.4)			
**Occupation**, ***n*** **(%)**‘						
Unemployed	6 (5.6)	4 (4.8)	2 (6.2)	-	-	-
Self-employed	26 (24.2)	6 (15.0)	20 (33.3)	4.765	2	0.144
Work for others	19 (17.7)	7 (19.5)	12 (19.7)			
Work in home	56 (52.3)	27 (67.5)	29 (47.5)			
**Marital status**, ***n*** **(%)**						
Single	35 (32.7)	9 (21.4)	26 (40)	4.225	1	0.040
Married	71 (66.4)	33 (78.6)	38 (58.5)			
Divorced	1 (0.9)	0 (0)	1 (1.5)	-	-	-
**BMI**, ***n*** **(%)**						
Underweight	4 (3.7)	0 (0.0)	4 (6.2)	-	-	-
Normal	27 (25.2)	1 (2.4)	26 (40.0)	32.706	2	< 0.0001
Overweight	34 (31.8)	11 (26.2)	23 (35.4)			
Obesity	42 (39.3)	30 (71.4)	12 (18.5)			
BMI, (kg/m^2^)	28.62 ± 6.26	32.95 ± 5.68	25.76 ± 4.82	−7.022	105	< 0.0001

Data are mean ± standard deviation for normally distributed or medians (interquartile ranges) for skewed parameters, or number (%). ^+^ Mann–Whitney U-test.

The clinical characteristics of the participants according to MetS status are shown in Table 3. The individuals with MetS were significantly more likely than the normal participants to have not aggressive OCD type (*p* = 0.009), with higher levels of abdominal obesity (*p* < 0.0001), TG (*p* < 0.0001), systolic blood pressure (*p* < 0.0001), fasting blood sugar (*p* < 0.0001), and lower levels of HDL-C (*p* < 0.0001). There was no significant difference among the age of OCD onset, duration of OCD treatment, OCD improvement based on the YBOCS score, and comorbidities in MetS and normal participants (Table 3).

**Table 3:** Comparing clinical characteristics between OCD patients with and without metabolic syndrome.

	**All patients (*n* = 107)**	**Metabolic Syndrome**		**x^2^/*t***	**df**	***p-*value**
		**Yes (*****n*** = **42)**	**No (*****n*** = **65)**			
Age of OCD Onset (years)	20.14 ± 8.35	21.31 ± 10.33	19.83 ± 6.75	1441.5^+^	-	0.624
Duration of OCD treatment (months)	47.58 ± 34.21	47.57 ± 26.17	47.59 ± 36.13	1457.5^+^	-	0.463
OCD type, *n* (%)
Washing	79 (73.8)	32 (76.2)	47 (72.3)	0.199	1	0.655
Symmetry	27 (25.2)	13 (31.0)	14 (21.5)	1.198	1	0.274
Hoarding	5 (4.7)	2 (4.8)	3 (4.6)	0.001^+^	1	0.972
Religious	20 (18.7)	8 (19.0)	12 (18.5)	0.006	1	0.939
Aggressive	21 (19.6)	3 (7.1)	18 (27.7)	6.830	1	0.009
Miscellaneous	21 (19.6)	9 (21.4)	12 (18.5)	0.142	1	0.706
Comorbidity, n (%)
GAD	31 (29.0)	14 (33.3)	17 (26.2)	0.639	1	0.424
PD	6 (5.6)	2 (4.8)	4 (6.2)	0.093^+^	1	0.760
Phobia	16 (15.0)	7 (16.7)	9 (13.8)	0.160	1	0.690
MDD	9 (8.4)	5 (11.9)	4 (6.2)	1.095^+^	1	0.310
BD	3 (2.8)	0 (0.0)	3 (4.6)	-	-	-
YBOCS
Total improvement	−15.09 ± 4.54	−15.43 ± 4.80	−14.83 ± 4.37	1218.5^+^	-	0.349
Obsessions improvement	−7.62 ± 2.79	−7.76 ± 3.11	−7.49 ± 2.58	1216.5^+^	-	0.339
Compulsions improvement	−7.47 ± 2.46	−7.67 ± 2.31	−7.34 ± 2.54	1271.0^+^	-	0.545
MetS component
AO, (cm)	104 (91–112)	111 (105.75–115)	93 (85.5–104)	2381.5^+^	-	< 0.0001
TG, (mmol/L)	140 (90–180)	180 (167–226)	100 (79–137.5)	2411.5^+^	-	< 0.0001
HDL-C, (mmol/L)	46 (39–60)	40 (35–45)	52 (45–60)	284.5^+^	-	< 0.0001
DBP, (mmHg)	75 (70–80)	77.5 (70–80)	75 (70–80)	1588.0^+^	-	0.134
SBP, (mmHg)	110 (100–120)	117.5 (110–130)	110 (100–115)	2002.5^+^	-	< 0.0001
FBS, (mmol/L)	95 (85–105)	106 (94.5–152.25)	90 (81–97.5)	2129.0^+^	-	< 0.0001

Data are mean ± standard deviation for normally distributed or median (interquartile range) for skewed parameters, or number (%). GAD, generalized anxiety disorder; PD, panic disorder; MDD, major depressive disorder; BD, bipolar disorder; AO, abdominal obesity; TG, triglyceride; HDL-C, high-density lipoprotein-cholesterol; DBP, diastolic blood pressure; SBP, systolic blood pressure; FBS, fasting blood sugar. ^+^ Mann–Whitney U-test; ^+^Fisher's exact test.

Table 4 shows risk factors predicting MetS in patients with OCD by the backward logistic regression model. All significant variables were entered into the model in step one. After four steps, age and BMI were retained in the model. There was no more difference between variance explanations (R^2^) in step one to step four. In line with expectations, an increase in age (OR = 1.05; CI = 1.01, 1.10) and BMI (OR = 1.36; CI = 1.19, 1.56) were predicted as having more odds of MetS (Table 4).

**Table 4:** Risk factors predicting metabolic syndrome in patients with OCD using the logistic regression model.

	**OR (95% CI)**	**Wald**	**df**	***p*-value**	**R^2^**
**Step 1**					
Age	1.07 (1.00–1.14)	3.692	1	0.055	0.529
Education	1.02 (0.89–1.17)	0.095	1	0.758	
Marital status	0.40 (0.09–1.86)	1.353	1	0.245	
Aggressive	0.29 (0.05- 1.76)	1.829	1	0.176	
BMI	1.38 (1.20–1.59)	21.062	1	< 0.0001	
Constant	0.00	17.289	1	< 0.0001	
**Step 2**					
Age	1.06 (1.01–1.12)	4.603	1	0.032	0.529
Marital status	0.41 (0.09–1.88)	1.303	1	0.254	
Aggressive	0.30 (0.05–1.77)	1.770	1	0.183	
BMI	1.38 (1.20- 1.58)	21.057	1	< 0.0001	
Constant	0.00	22.961	1	< 0.0001	
**Step 3**					
Age	1.04 (1.00–1.09)	3.612	1	0.057	0.518
Aggressive	0.29 (0.05- 1.69)	1.903	1	0.168	
BMI	1.36 (1.19- 1.56)	20.550	1	< 0.0001	
Constant	0.00	22.335	1	< 0.0001	
**Step 4**					
Age	1.05 (1.01- 1.10)	5.902	1	0.015	0.501
BMI	1.36 (1.19- 1.56)	20.429	1	< 0.0001	
Constant	0.00	24.148	1	< 0.0001	

BMI, body mass index; OR, odd ratio; CI, confidence interval.

## Discussion

This study is the first Iranian study to investigate MetS in patients with OCD. In this study, 39.2% of the patients with OCD met the MetS diagnostic criteria which was higher than the frequencies reported in two Italian clinical (21%, 19.8%) (24, 25) and one Swedish cohort (25%) (22) studies. The reported prevalence of MetS in all the studies was higher than the reported prevalence of MetS in their own country's general population (22, 24, 25). In Iranian studies, the frequency of MetS in schizophrenia and bipolar disorder was between 23.9 and 27.4% (30–32). It was lower than the frequency of MetS in patients with OCD in our study.

People with major psychiatric disorders, such as schizophrenia, bipolar disorder, and depression, are at higher risk for MetS (19, 33, 34). In line with the results of other clinical studies, the present study showed that MetS is present with significant frequency in patients with OCD similar to other major psychiatric disorders. The high prevalence of MetS in patients with OCD and its adverse health consequences, calls for further studies. The results of cohort studies (22, 23, 35) have also shown that cardiovascular and metabolic diseases are a serious threat to health and the cause of increased mortality and premature death in patients with OCD (35). Due to the relationship among MetS, diabetes, and cardiovascular disease (36), appropriate medical interventions to improve the metabolic status of patients with OCD seem to be necessary.

In our study, the prevalence of MetS that increased with age was expected. In addition, there was a negative relationship between the level of education and MetS. Unlike Albert et al. (24) study, we found a negative relationship between the level of education and MetS. One explanation is the higher education level and a more minor standard deviation in that study. Higher education levels may lead to more attention to health issues and personal self-care (37, 38).

MetS was more common in married people. It may be related to their diet, lifestyle, and level of physical activity (39). Physical activity reduces the risk of metabolic syndrome or general health conditions. There has been an idea that physical activity may reduce obsessive-compulsive symptoms in patients with OCD. Several studies demonstrated that exercise reduced obsessive-compulsive symptoms, while the only randomized controlled trial did not show its efficacy (40). Little physical activity due to the involvement of patients in time-consuming OC activities along with other risk factors may increase the risk of cardiovascular and metabolic diseases. Behavioral tasks designed with appropriate physical activity may reduce OC symptoms and the frequency of MetS.

Unlike two Italian studies (24, 25), we found no association between the duration of OCD and MetS. Interestingly, in a Swedish cohort study, individuals with OCD who had a longer treatment duration had significantly lower risks of metabolic and cardiovascular disorders (22).

We found a significant relationship between BMI and MetS. This finding has been reported in many studies (41). Hypertension was the lowest, and abdominal obesity was the most common component of the MetS among patients. In the study by Albert et al. (24), hypertension followed by abdominal obesity was the most common component of MetS. In a study by Aguglia et al. (25), hyperlipidemia and hypertension had the highest frequency.

Guilan province is an agricultural hub, and the daily diet of most people, especially in rural areas, is rice. In cold seasons of the year, the physical activities of people dramatically decrease. High-calorie intake and reduced physical activity may thus lead to increased fat storage. In our study, the prevalence of overweight/obesity with a BMI of 25 or higher and abdominal obesity were 71 and 68.2%, respectively. Contrary to the results of some studies, we found no association between contamination/washing symptom and MetS (42, 43). The absence of aggressive symptoms was associated with MetS in our study. Due to the small sample size of this study, it is difficult to explain the importance of this finding. Longitudinal studies with bigger sample sizes are recommended.

Among the studied variables, only one demographic variable (age) and one medical variable (BMI) could predict the MetS, while none of the clinical variables could predict the syndrome. This finding has been reported in the previous OCD study (24) and other MetS studies in patients with bipolar disorder and depressive disorder (19, 33), other medical disorders (8), and community studies (44). We can conclude that age and BMI are the strongest predictors of MetS in the general population and psychiatric patients, including patients with OCD. In this study, several psychiatric clinical variables were examined. The results showed that these variables have no significant relationship with the MetS. Due to the small number of studies, sufficient explanation is needed to further studies.

In the present study, all patients received medication, while in the Albert et al.' study (24), 20% of patients did not receive any medication. In total, 12% of our patients took SGAs, while 40% of patients in the Italian study were given these drugs. The OCD severity in our sample was in the range of severe and very severe. Most of the patients had already seen other therapists and had received pharmacological and non-pharmacological treatments. At the time of the final evaluation, patients were on established treatment. The duration of treatment and age of onset was not associated with MetS in our study. It means that exposure duration did not increase the risk of MetS. In interpreting this finding, we should consider the treatment strategies used in each clinic. In case of inadequate response to SSRIs, we added risperidone or aripiprazole. We did not add lithium or valproate to augment the treatment response. The previous studies did not support the effectiveness of adding mood stabilizers (24, 45). Glutamatergic agents were added to SSRIs (more memantine and three cases with lamotrigine) in 23% of our cases who were unresponsive to augmentation with SGAs. Using SGAs is associated with worsening MetS (46). We expected to see a more impaired metabolic status in augmented cases, but unexpectedly the prevalence of MetS in augmented and non-augmented cases was not significantly different. Compared with patients who were not taking SSRIs patients with OCD, those who were taking higher doses of SSRIs and who had a longer duration of treatment had lower risks of metabolic and cardiovascular complications, regardless of whether they were also taking SGAs (35). One explanation is that the treatment brings about desirable changes in patients' lifestyles, physical activity, and time management. It might slightly moderate the adverse metabolic effects of adding drugs. There was no association between OCD-comorbidity and MetS which is in line with the results of the Swedish cohort study (22, 35). Three patients with bipolar disorder were included in our study. The number of bipolar patients in Albert et al. (24) study was significantly more than in our study. In Aguglia et al. (25) study, all patients with bipolar disorder were excluded. The frequency of the use of mood stabilizers or SGA in different studies can influence the reported frequency of MetS (35). Our study had some limitations. All patients and their families participated in psychoeducation sessions. Some patients also received cognitive behavioral therapy at the same time. We did not consider psychotherapy as a variable. In addition, we had to use medical records and history to diagnose OCD and its comorbidities in some patients, which could reduce our diagnostic accuracy. The small sample size, cross-sectional design, lack of a control group, and the limited number of comorbidities were the other limitations of this study.

## Conclusion

The results of this study were consistent with the results of other clinical cross-sectional and cohort studies that reported the poor health status of patients with OCD. A large number of patients with OCD are affected or are at a high risk of developing MetS. These patients require planned medical care and regular evaluation of metabolic status and cardiovascular risk factors along with usual OCD treatments.

## Data availability statement

The original contributions presented in the study are included in the article/supplementary material, further inquiries can be directed to the corresponding author.

## Ethics statement

The studies involving human participants were reviewed and approved by the Ethics Committee of Guilan University of Medical Sciences. The patients/participants provided their written informed consent to participate in this study.

## Author contributions

All authors listed have made a substantial, direct, and intellectual contribution to the work and approved it for publication.
